# Brain Development and Akt Signaling: the Crossroads of Signaling Pathway and Neurodevelopmental Diseases

**DOI:** 10.1007/s12031-016-0872-y

**Published:** 2016-12-26

**Authors:** Long Wang, Kai Zhou, Zhi Fu, Di Yu, Hesuyuan Huang, Xiaodong Zang, Xuming Mo

**Affiliations:** Department of Cardiothoracic Surgery, Children’s Hospital of Nanjing Medical University, Nanjing, 210008 China

**Keywords:** Brain development, PI3K-Akt-mTOR signaling pathway, Neurodevelopmental diseases, Megalencephaly, Microcephaly

## Abstract

Neurodevelopmental biology, coupled with the application of advanced histological, imaging, molecular, cellular, biochemical, and genetic approaches, has provided new insights into these intricate genetic, cellular, and molecular events. During telencephalic development, specific neural progenitor cells (NPCs) proliferate, differentiate into numerous cell types, migrate to their apposite positions, and form an integrated circuitry. Critical disturbance to this dynamic process via genetic and environmental risk can cause neurological disorders and disability. The phosphatidylinositol-3-OH kinase (PI3K)-Akt-mammalian target of rapamycin (mTOR) signaling cascade contributes to mediate various cellular processes, including cell proliferation and growth, and nutrient uptake. In light of its critical function, dysregulation of this node has been regarded as a root cause of several neurodevelopmental diseases, such as megalencephaly (“big brain”), microcephaly (“small brain”), autism spectrum disorders, intellectual disability, schizophrenia, and epilepsy. In this review, particular emphasis will be given to the PI3K-Akt-mTOR signaling pathway and their paramount importance in neurodevelopment of the cerebral neocortex, because of its critical roles in complex cognition, emotional regulation, language, and behaviors.

## Introduction

The mammalian neocortex is a complicated, tightly orchestrated, and six-layered construction that contains various neuronal subtypes and glias. The diverse neurons and glias of the central nervous system (CNS) are produced by a small, heterogeneous population of neural progenitor cells (NPCs) that undergo transcriptional changes to sequentially specific distinct cell fates, guided by temporal cell extrinsic and intrinsic cues. Astrocytes and oligodendrocytes (OLs), the major glial sub-lineages in the CNS, play key roles in telencephalic development and homeostatic maintenance with increasing brain complexity, controlling various aspects of neurodevelopment and diseases (Gallo & Deneen 2014; Zuchero & Barres 2015).

PI3K-Akt-mTOR cues regulate various cellular functions, including nutrient uptake, cell proliferation, growth, autophagy, apoptosis, and migration (Hennessy et al. 2005; Yu & Cui 2016). In the absence of extracellular stimulators, Akt is cytoplasmic and inactive (Alessi et al. 1996). Upon phosphor-activated by PI3K, Akt is recruited to the plasma membrane through binding of its pleckstrin homology (PH) domain to phosphatidylinositol-1,4,5-trisphosphate (PIP3), which is produced by PI3K (Alessi et al. 1997). Translocation of Akt enables phosphorylation of Thr308 on its activation loop by membrane-localized phosphoinositide dependent kinase 1 (Pdk1) (Alessi et al. 1997; Stokoe et al. 1997). Full activation of Akt requires phosphorylation of Ser473, which lies in a C-terminal hydrophobic motif (HM) of Akt, by the rapamycin-insensitive mTORC2 (Sarbassov et al. 2005). Akt further stimulates mTORC1 through directly or indirectly suppression of TSC1/2 to abolish its inhibition of Rheb1, thereby stimulating mTORC1 (Laplante & Sabatini 2012). Another important negative regulator of the PI3K-Akt-mTOR signaling pathway is Pten (phosphatase and tensin homolog). Through its lipid phosphatase activity, Pten dephosphorylates PIP3 to PIP2 and precisely counteracts the kinase function of PI3K and subsequent activation of downstream Akt-mTORC1 signaling (Song et al. 2012). The best-defined downstream targets of Akt-mTORC1 are the p70 ribosomal S6 protein kinases 1 and 2 (S6K1/2) (Fenton & Gout 2011) and the eukaryotic initiation factor 4E-binding proteins (4E-BPs), which are in direct response to mTORC1 activation to initiate translation (Ma & Blenis 2009).

**Fig. 1:**
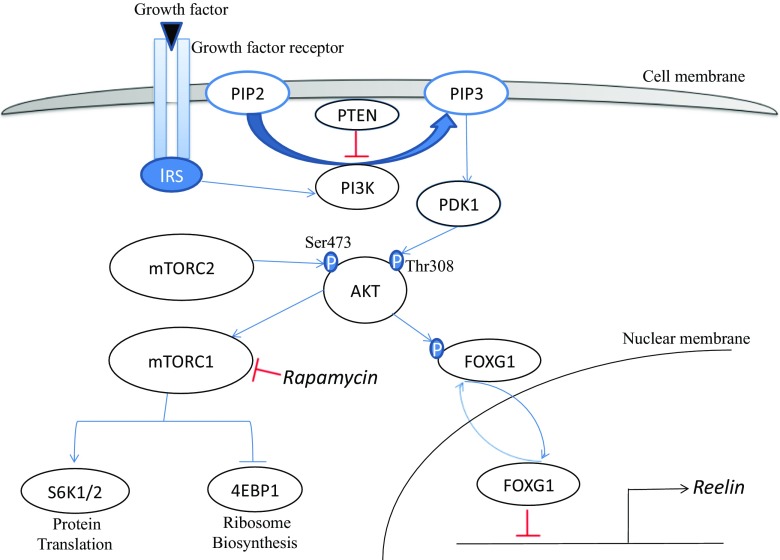
Molecular mechanisms of PI3K-AKT-mTOR signaling cascade related neurodevelopmental diseases

Dysfunction of PI3K-Akt-mTOR cascade has been recognized as the root cause of both neurodevelopmental and neuropsychiatric diseases with distinct clinical phenotypes, such as autism spectrum disorder, epilepsy, brain injury, and a spectrum of developing brain malformations (Crino 2016; Wang et al. 2015; Yang & Mo 2016; Yu et al. 2015). In this review, we discuss the functions of this pathway during embryonic forebrain development, with a particular focus on the putative molecular mechanisms underlying these functions (Fig. 1).

## Dysregulation of Akt3 Is Associated with Neurodevelopmental Disorders

Akt (also called protein kinase B or Pkb) is a member of the serine/threonine protein kinase AGC family and has three isoforms Akt1/Pkbα, Akt2/Pkbβ, and Akt3/Pkbγ. Akt, as a central node, is a positive regulator of several signaling pathways including cell proliferation, growth, survival, and metabolism across many species (Engelman et al. 2006; Hennessy et al. 2005; Hou & Klann 2004; Manning & Cantley 2007). Although the three Akt isoforms show high homology and share similar structures, mouse genetics have demonstrated that they play not only over-lapping but also specialized roles in development and physiology (Bae et al. 2003; Chen et al. 2010; Cho, Mu et al. 2001a; Cho, Thorvaldsen et al. 2001b; Yun et al. 2008).

Akt3 expression in the human fetal and mouse adult brain is higher than its expression in any other tissue sampled, whereas Akt1 and Akt2 show comparable to or lower levels of fetal brain expression than those seen in other tissues (Easton et al. 2005; Wu et al. 2009). Moreover, Akt3 is expressed at higher levels than Akt1 and Akt2 in the human fetal and mouse adult brain (Easton et al. 2005; Wu et al. 2009). Therefore, Akt3 was the predominant isoform and present in all areas of the adult mouse brain, representing about one half of the total Akt protein in adult brains (Easton et al. 2005; Wu et al. 2009).

Although specialized substrate and function of protein kinases might be attributed to their tissue-specific expression, the virtually ubiquitous localization of many critical kinases, including Akt, makes this an unlikely general mechanism. An appealing area of Akt studies is also to define their isoform-specific roles. Immunostaining using an antiserum that recognizes all three phospho-activated forms of Akt (p-Akt) exhibits widespread p-Akt localization in the developing cortex, with remarkable enrichment in neural progenitor cells in the ventricular zone, suggesting Akt’s primary role in brain development (Poduri et al. 2012). Further support for the distinct role of Akt3 in controlling brain size firstly comes from animal studies. Two independent mouse Akt3 knockout models show selective reduction in brain size (Easton et al. 2005; Tschopp et al. 2005), whereas mice with an activating mutation of Akt3 show enlarged brain size and increased corpus callosum thickness (Tokuda et al. 2011). Unlike the Akt3 isoform, mice lacking single Akt1 and Akt2 show smaller size of multiple organs and a diabetes-like syndrome, respectively (Cho et al. 2001a; Cho et al. 2001b). Deletions of chromosome 1q42-q44 (encompassing the *AKT3* gene) in human genome have been reported in a variety of developmental aberrations of the brain, including agenesis of the corpus callosum (ACC) and microcephaly. Several clinical studies demonstrate that haploinsufficiency of AKT3 in this region causes microcephaly and ACC (Boland et al. 2007; Wang et al. 2013). However, the cellular and molecular mechanisms of the disease and the strategy for therapies remain poorly defined.

Disturbances of cortical development at various critical stages—such as neural proliferation, migration, and orchestration—lead to representative malformations of cortical development (MCD) (D’Gama et al. 2015; Jansen et al. 2015; Lee et al. 2012; Riviere et al. 2012; Striano & Zara 2012). MCD are progressively recognized as a critical cause of neurodevelopmental delay, intellectual disability, ASD, and especially clinically intractable “catastrophic” epilepsy (Striano & Zara 2012). The most severe type of the spectrum is hemi-megalencephaly (HME), characterized by enlargement of most or all of one entire cerebral hemisphere, typically causing a medically severe pediatric epilepsy that requires surgical resection (Flores-Sarnat et al. 2003). Post-zygotic somatic activation of *AKT3* is found in a wide range of brain diseases, including megalencephaly (“big brains”) and HME. De novo germline 1q43q44 (encompassing the *AKT3* gene) trisomy has been reported in megalencephaly (Jansen et al. 2015). Somatic chromosome 1q43q44 (encompassing the *AKT3* gene) tetrasomy and a gain of function mutation in *AKT3* (c.49G/ A, creating p.E17K), have been reported in HME (Poduri et al. 2012; Wang et al. 2013). In contrast, most strikingly though, the somatic *AKT3* mutation identified is highly paralogous to the common E17K mutations in *AKT1* and *AKT2*, associated with Proteus syndrome, another multisystem overgrowth disorder, and hypoglycemia and left-sided overgrowth, respectively (Hussain et al. 2011; Lindhurst et al. 2011). Introducing the focal MCD-causing *AKT3* ^E17K^ mutation into the mouse brain causes impaired hemispheric architecture and electrographic seizures (Baek et al. 2015). Mutant *AKT3* ^E17K^-expressing NPCs showed dysregulation of Reelin, which leads to a non-cell autonomous migration defect in neighboring cells, due at least in part to transcriptional de-repression of Reelin (Baek et al. 2015). The forkhead box (FOX) transcription factors has been established to function as transcriptional repressors, but after phosphorylation by AKT, FOXG1 translocates to the cytoplasm, thereby attenuating its transcriptional repression of Reelin (Brunet et al. 1999; Manning & Cantley 2007). Therapies aimed at either suppressing downstream AKT pathway by rapamycin or Reelin inactivation restored disrupted neuronal migration (Baek et al. 2015). These findings demonstrate a central AKT3-FOXG1-Reelin pathway in focal MCD, which also benefit to define how a mutation in just a few fraction of cells could perturb the gross organization of the entire hemisphere and elicit such devastating defects in brain development (Baek et al. 2015).

## PI3K-AKT-mTOR Signaling in Neurodevelopment

A growing number of developmental brain malformations, characterized by altered cerebral architecture, abnormal neuronal morphology, and, often, intractable epilepsy, has recently been associated with novel mutations in genes encoding components of the Akt node (Striano & Zara 2012). Megalencephaly syndromes are probably genetically mosaic diseases caused by gain of function mutations in the PI3K–Akt3–mTOR pathway (D’Gama et al. 2015; Flores-Sarnat et al. 2003; Jansen et al. 2015; Lee et al. 2012; Riviere et al. 2012; Striano & Zara 2012). Recently, germline and somatic point mutations in *AKT3*, *PIK3R2*, and *PIK3CA* have been detected in the megalencephaly-related syndrome, and somatic gain of function point mutations in *AKT3*, *PIK3CA*, and *MTOR* has also been detected in HME, the most severe type of megalencephaly (Baek et al. 2015; Jansen et al. 2015; Lee et al. 2012; Nakamura et al. 2014; Poduri et al. 2012; Riviere et al. 2012). Sequencing at the single cell level identified a mutation burden in both neuronal and non-neuronal cells, denoting that mutations occur mainly in NPCs (Evrony et al. 2012; Poduri et al. 2013).

Conventional and conditional ablation of key components of the PI3K-Akt-mTOR pathway in mouse, such as Pten, Pdk1, Tsc1/2, mTOR, and Raptor (Costa-Mattioli & Monteggia 2013; Huber et al. 2015; Lipton & Sahin 2014; Zhou & Parada 2012), contributes to mechanistic researches and development of therapies for these devastating disorders. The brains deficient for *Pdk1*, *Akt*, *mTOR*, and *Raptor* all exhibit microcephaly (Costa-Mattioli & Monteggia 2013; Huber et al. 2015; Lipton & Sahin 2014; Zhou & Parada 2012). The disruption of mTOR resulted in aberrant cell cycle progression of NPCs in the developing forebrain and thereby disruption of progenitor self-renewal (Ka et al. 2014). Accordingly, genesis of intermediate progenitors and post-mitotic neurons were markedly prohibited (Ka et al. 2014). The brains deficient for raptor, essential for mTORC1 activity, exhibit a small brain starting at E17.5, which is the outcome of a decline in cell number and size (Cloetta et al. 2013).

Loss of *Pten*, leading to amplification of the AKT-mTOR signaling pathway, is a risk factor for macrocephaly, ASD, and glioma (Fraser et al. 2004; Li et al. 2002). Conditional elimination of *Pten*, causing hyperactivation of downstream Akt-mTOR pathway in the mouse CNS, has elucidated multiple roles in brain development and maintenance (Fraser et al. 2004; Groszer et al. 2001; Li et al. 2002; Marino et al. 2002; Yue et al. 2005). *Pten* deletion in NPCs resulted in its elevated proliferation and self-renewal in vitro and in vivo (Groszer et al. 2001), whereas *Pten* disruption in premature neurons caused hypertrophy without alteration on NPC proliferation (Fraser et al. 2004). *Pten* haploinsufficiency (*Pten* ^+/−^) leads to a dynamic trajectory of brain overgrowth and altered scaling of neural cells, with an elevation of beta-catenin signaling (Chen et al. 2015). A heterozygous mutation in beta-catenin, itself a risk gene for microcephaly and ASD, inhibits cerebral overgrowth in *Pten* ^+/−^ mice, which provide a new perspective that Pten and beta-catenin signaling act in conjunction to control neural cell number and normal brain growth trajectory (Chen et al. 2015).

Together, the emerging consensus is that elevation of the PI3K-AKT-mTOR signaling pathway leads to enhanced proliferation of progenitors, neuronal hypertrophy, and excessive dendritic branching, whereas suppression exhibits the opposite consequences (Costa-Mattioli & Monteggia 2013; Huber et al. 2015; Lipton & Sahin 2014; Zhou & Parada 2012).

## Conclusions

Germline or widespread somatic mutations of PI3K-AKT-mTOR signaling networks may elicit overt brain architecture defects, whereas subtle, somatic, or cell type-specific mutations may lead to localized and restricted abnormalities. The severity and medical characteristics of neurodevelopmental diseases may be determined partially by the stage at which the mosaicism occurs relative to the distinct period of neurogenesis and gliogenesis, and which types of neural cells are affected. Therefore, more detailed researches are needed to decipher the cell type-specific effects of mosaic mutation and to determine which type of pathologies is attributed to specialized neural malfunction (Cai et al. 2014; Evrony et al. 2012; Poduri et al. 2013). Mouse models that manipulate the individual component of the PI3K-AKT-mTOR pathway by genetic deletion in distinct neural cell types recapitulate the characteristic pathogenesis of neurodevelopmental diseases and contribute to define the underlying mechanisms and develop therapies for these catastrophic disorders.
